# Unsupervised machine learning model for detecting anomalous volumetric modulated arc therapy plans for lung cancer patients

**DOI:** 10.3389/fdata.2024.1462745

**Published:** 2024-10-03

**Authors:** Peng Huang, Jiawen Shang, Yuhan Fan, Zhihui Hu, Jianrong Dai, Zhiqiang Liu, Hui Yan

**Affiliations:** Department of Radiation Oncology, National Cancer Center/National Clinical Research Center for Cancer/Cancer Hospital, Chinese Academy of Medical Sciences and Peking Union Medical College, Beijing, China

**Keywords:** volumetric modulated arc therapy, AutoEncoder, anomaly detection, radiotherapy, lung cancer

## Abstract

**Purpose:**

Volumetric modulated arc therapy (VMAT) is a new treatment modality in modern radiotherapy. To ensure the quality of the radiotherapy plan, a physics plan review is routinely conducted by senior clinicians; however, this process is less efficient and less accurate. In this study, a multi-task AutoEncoder (AE) is proposed to automate anomaly detection of VMAT plans for lung cancer patients.

**Methods:**

The feature maps are first extracted from a VMAT plan. Then, a multi-task AE is trained based on the input of a feature map, and its output is the two targets (beam aperture and prescribed dose). Based on the distribution of reconstruction errors on the training set, a detection threshold value is obtained. For a testing sample, its reconstruction error is calculated using the AE model and compared with the threshold value to determine its classes (anomaly or regular). The proposed multi-task AE model is compared to the other existing AE models, including Vanilla AE, Contractive AE, and Variational AE. The area under the receiver operating characteristic curve (AUC) and the other statistics are used to evaluate the performance of these models.

**Results:**

Among the four tested AE models, the proposed multi-task AE model achieves the highest values in AUC (0.964), accuracy (0.821), precision (0.471), and *F*1 score (0.632), and the lowest value in FPR (0.206).

**Conclusion:**

The proposed multi-task AE model using two-dimensional (2D) feature maps can effectively detect anomalies in radiotherapy plans for lung cancer patients. Compared to the other existing AE models, the multi-task AE is more accurate and efficient. The proposed model provides a feasible way to carry out automated anomaly detection of VMAT plans in radiotherapy.

## 1 Introduction

Machine learning (ML) is an interdisciplinary field based on mathematics, statistics, and data processing. It is a specific type of artificial intelligence that collects data from applications for training (Ethem, 2020). ML is used in many real-world applications and is essential in several fields such as image recognition (Chan et al., 2020), image segmentation (Chen et al., 2022), natural language processing (Wu et al., 2020), and fraud detection (Chalapathy and Chawla, 2019). In the healthcare sector, ML is mainly used in medical record analysis and disease forecasts (Shehab et al., 2022). ML is successfully adopted by a wide range of medical applications, such as COVID-19 detection (Rani et al., 2022a; Minaee et al., 2020), multi-organ segmentation (Asgari Taghanaki et al., 2021; Azad et al., 2024), and bone suppression (Rani et al., 2022b; Yang et al., 2017). In this study, we explore its potential in detecting anomalies from treatment plan records in radiotherapy.

Radiotherapy has been an indispensable component for cancer patient treatment. Currently, ~60% of cancer patients receive radiotherapy for definitive, adjuvant, or palliative treatment. Furthermore, the percentage of cancer survivors treated by radiotherapy alone or combined with other treatment modalities, such as surgery and chemotherapy, is close to 40% (Du et al., 2022). Modern medical linear accelerators can deliver higher radiation doses to tumors while minimizing exposure to the surrounding organs at risk. This allows for significant destruction of tumor tissue tissue within the target volume, while significantly protecting surrounding healthy tissue from irradiation (Gardner et al., 2019). As the complex process heavily relies on ionized radiation, a highly accurate treatment plan is required to ensure the prescribed dose is safely delivered to the patient. In many reported accidents, even a small error or mistake could result in serious issues for the patient under radiotherapy (Du et al., 2022).

As planning and delivering doses to patients is a complex process in modern radiotherapy, it is crucial to secure the highest quality control over the whole treatment process. For this purpose, routine daily physics plans and chart reviews are required and conducted by senior medical physicists. While human-led plan reviews are effective and reliable, they can also be inefficient and error-prone (Ganesh, 2014). The contents of a physics plan review are mostly based on clinical guidelines such as AAPM TG 275 (Ford et al., 2020) and MPPG (Xia et al., 2021). They are designed mainly for traditional radiotherapy techniques such as three-dimensional conformal radiotherapy (3D-CRT) and intensity-modulated radiotherapy (IMRT) and include items such as simulation imaging, dose prescription, treatment planning, and mechanical parameters (Yang et al., 2012). For new treatment techniques, such as volumetric modulated arc therapy (VMAT), existing guidelines (Palta et al., 2008) previously designed for IMRT are not appropriate and should be updated.

Automated methods were introduced to assist the physics plan review process in recent years (Gopan et al., 2016). Most of them are rule-based applications and automate the checking process in the physics plan review. In a clinical setting, these methods are implemented in the oncology information system and run as background processes. A semi-automatic system was proposed to assist the automatic inspection of the treatment plan by Dewhurst et al. (2015). An automatic tool to check and compare radiotherapy plans was developed by Covington et al. (2016). To perform intra-plan and inter-plan reviewing automatically, software was developed by Furhang et al. (2009). To verify the integrity of the treatment plan automatically, dynamic scripts were implemented by Yang and Moore (2012). With the emergence of automated tools, the accuracy and efficiency of physics plan reviews significantly improved.

Anomaly detection is a prominent area of research in computer vision and pattern recognition (Hojjati et al., 2024). There are many methods to detect anomalies using various machine learning methods, such as principal component analysis (PCA) and *K*-means clustering. In recent years, deep-learning neural networks have demonstrated unprecedented results over traditional machine learning methods (Pang et al., 2021). As a popular network, AutoEncoder (AE) has been widely used in many industries, including shape representation (Chalapathy and Chawla, 2019; Pimentel et al., 2014), credit fraud detection (Misra et al., 2020; Fanai and Abbasimehr, 2023), and network attack monitoring (Song et al., 2021; Lopes et al., 2022). Recently, AE has been introduced in radiotherapy for modeling organ motion (Mezheritsky et al., 2022), detecting rare machine events (Dou et al., 2022), and conducting patient-specific QA (Wang et al., 2020).

**Figure 1:**
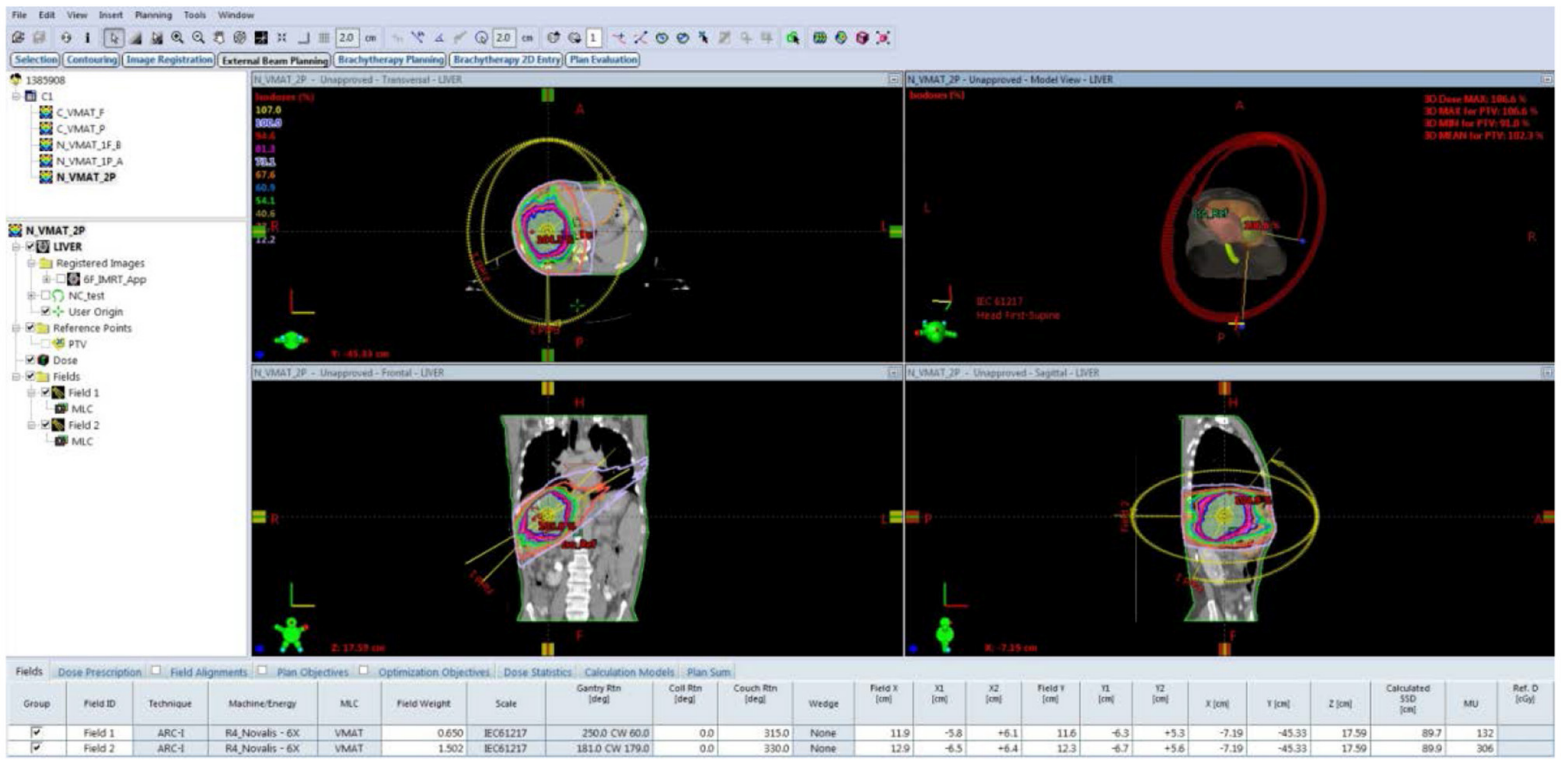
Treatment planning interface of A VMAT plan consisting of two arcs for liver cancer.

There are several AE models developed for anomaly detection in automatic plan review, but most of them focus on simple plan configurations, such as 3D-CRT and IMRT (Huang et al., 2023a; Azmandian et al., 2007; Kisling et al., 2020). In these applications, a few feature parameters of a plan, including segment number, collimator positions, gantry angle, and monitor unit (MU), were extracted from the treatment plan and used to train the model (Huang et al., 2023b). As shown in Table 1, a set of feature parameters were extracted to represent an IMRT plan. Recently, VMAT replaced 3D-CRT and IMRT as the main treatment modalities in radiotherapy. Unlike traditional techniques, a VMAT plan consists of hundreds of gantry angles in a treatment, as shown in Figure 1. For catching the speed of gantry rotation and MLC leaf movement, the leaf positions during continuous gantry rotation are optimized for the best delivery efficiency (Otto, 2008). As there are thousands of parameters used in a VMAT plan, identifying a set of salient features is challenging.

**Table 1:** Summary of the original features obtained from IMRT plans.

**Feature**	**Description**	**Number of features**	**Type**	**Unit**
Segment	Number of segments of the field	2	Integer	Number
SSD	Source to skin distance	4	Float	cm
Coll_x1_	Collimator position in *x*1 direction	4	Float	cm
Coll_x2_	Collimator position in *x*2 direction	4	Float	cm
Coll_y1_	Collimator position in *y*1 direction	4	Float	cm
Coll_y2_	Collimator position in *y*2 direction	4	Float	cm
*C*_θ_	Angle of collimator	4	Integer	Degree
*G*_θ_	Angle of gantry	4	Integer	Degree
Meter set	MU per field	4	Float	MU
Dose	Dose per fraction	1	Float	cGy

SSD, source to surface distance; Coll, collimator; MU, monitor unit.

In this study, we proposed to create an aperture-based feature map to represent the shape of the treatment beam at each gantry angle. Based on the feature map, a multi-task AutoEncoder (multi-task AE) model is built to detect anomalous plans by assessing the magnitude of the reconstruction error. This study presents a novel way to perform automatic plan review in VMAT radiotherapy. The rest of the paper is organized as follows: In Section 2, the method to generate a feature map from the VMAT plan is described, followed by an introduction to multi-task AE model learning and evaluation. In Section 3, the effect of the distance metrics on model performance is evaluated, and the proposed AE model is compared to the other three existing AE models. In Section 4, the merits and limitations of the proposed method are discussed.

## 2 Materials and methods

### 2.1 Aperture-based feature map

**Figure 2:**
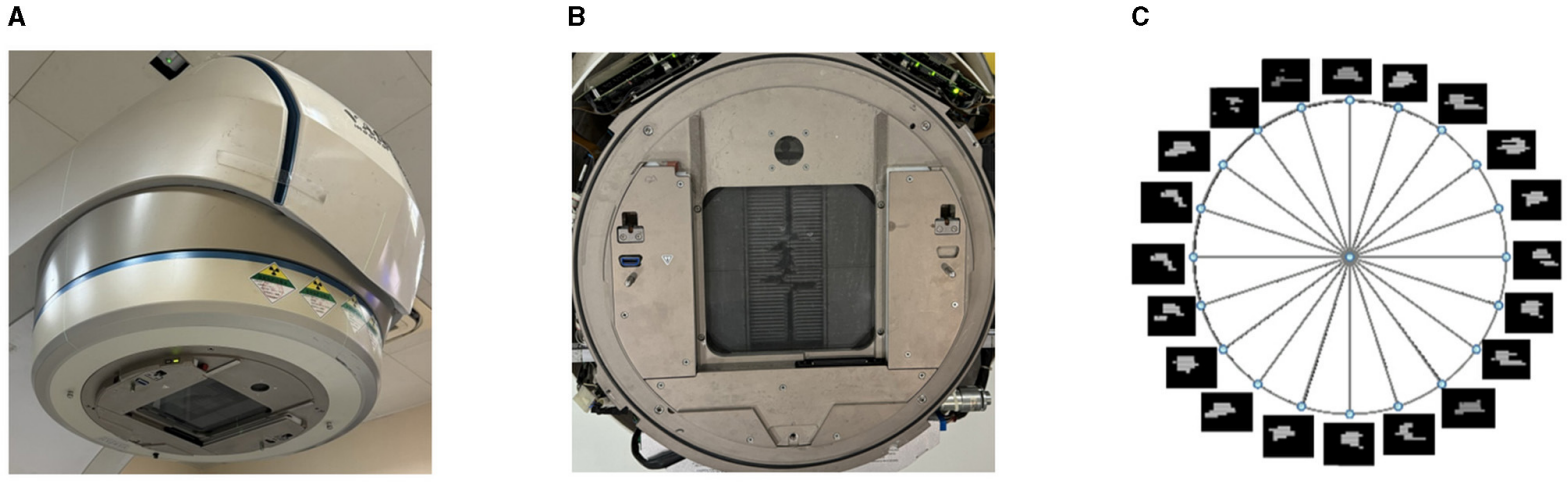
Example of machine head with MLC, beam aperture, and intensity maps of beams in a VMAT plan. **(A)** The MLC attached to the head of a radiotherapy machine. **(B)** The aperture created by MLC leaves. **(C)** The intensity maps of beams in a VMAT plan.

The multi-leaf collimator (MLC) consists of a set of thin tungsten leaves attached to a carriage on the treatment machine head, as shown in Figure 2A. The multiple leaves can be used to shape the aperture of the beam as shown in Figure 2B. Volumetric modulated arc therapy (VMAT) usually involves 100 beams along a 360°arc, with intervals of 2–4°, as shown in Figure 2C. For each beam or control point (CP) along the arc, the leaf positions and dose are determined by the treatment planning software. The leaf positions at a CP in a VMAT plan are constrained by the limitation of leaf speed.

**Figure 3:**
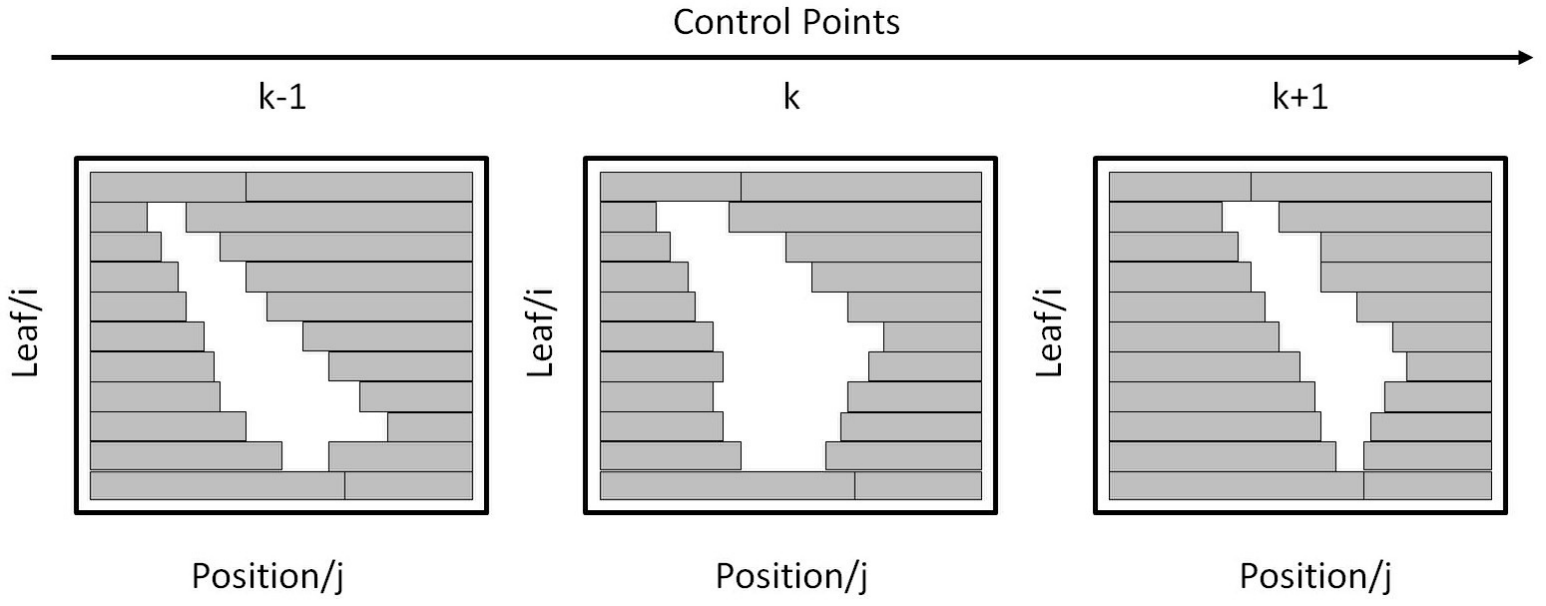
Apertures formed by MLC leaves at three consecutive CPs.

For a beam aperture at *k*-th CP, its shape can be defined by the leaf index *i* and position index *j* on a coordinate grid as shown in Figure 3. The real leaf width varies from thinner one (such as 0.25 mm) in the middle to thicker one (such as 0.50 mm) at the edge for different machines. For digitization, the leaf width is re-sampled to a finer resolution. For example, if the field width is 300 mm and the resolution is set to 0.1 mm, then the maximum leaf index *N*_*L*_ is 300/0.1 = 3,000. The leaf position is determined using its position index multiplied by the step size. As the step size is varied for different machines, for simplification, the step size is re-sampled to the finer resolution. For example, if the field height is 400 mm and the resolution is set to 0.1 mm, then the maximum position index *N*_*P*_ is 400/0.1 = 4,000.

At each CP, the set of all leaves and their positions form a region. The corresponding aperture at *k*-th CP, *A*_*k*_, is defined as all pairs of (*i, j*) falling within this region.


(1) $$\begin{array}{l} A_{k}\left(i,j\right)=\left\{ \begin{array}{ll} 1,\; & \left(i,j\right)\in Region_{k}\\ 0,\; & else \end{array}\right. \end{array}$$

**Figure 4:**
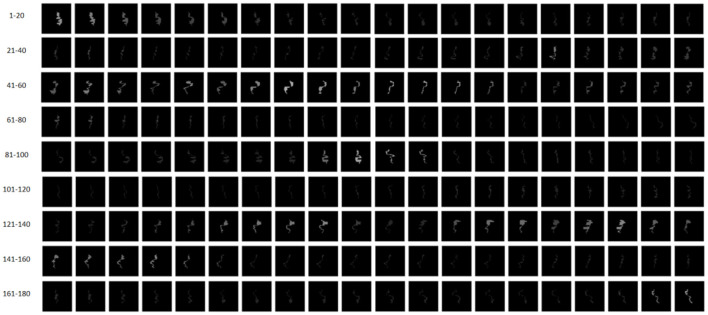
Intensity maps corresponding to 180 CPs of a VMAT plan. CPs 1–90 and CPs 91–180 belong to Arc 1 and Arc 2, respectively.

For the aperture *A*_*k*_, its intensity map of *I*_*k*_ is computed as


(2) $$\begin{array}{l} I_{k}\left(i,j\right)=D_{k}A_{k}\left(i,j\right), \end{array}$$

where *D*_*k*_ represents the dose (cGy) or monitor unit at the *k*-th CP. For simplification, the intensity map is re-normalized to a uniform image resolution with a pixel size of 0.1 by 0.1 mm. The intensity map is float-point two-dimensional matrix. Compared to the features shown in Table 1, the intensity map is a combination of the geometrical and dosimetric features. As a result, the intensity maps corresponding to 180 CPs of a VMAT plan are shown in Figure 4.

### 2.2 Multi-task AE model

**Figure 5:**
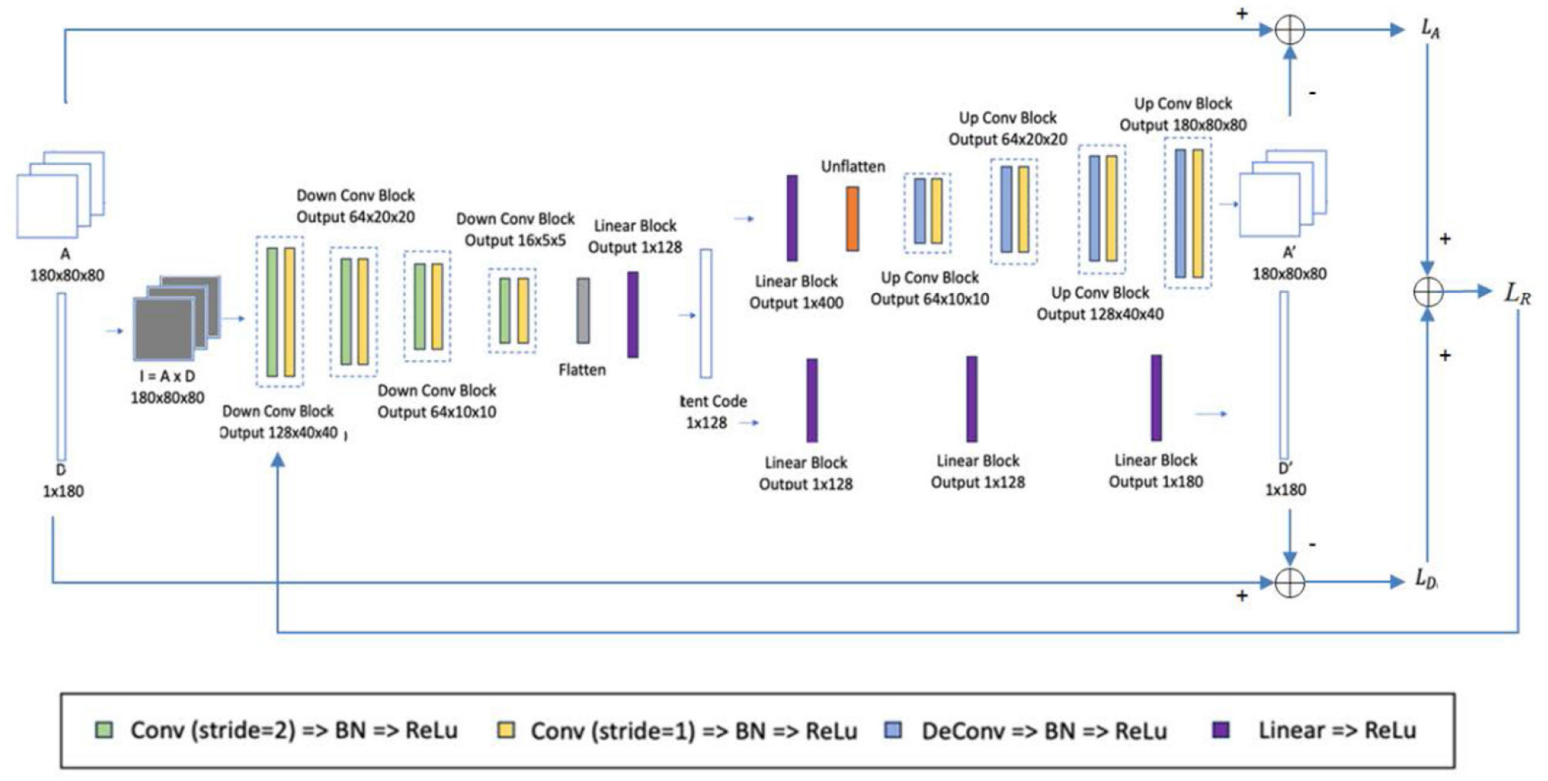
Network architecture of the multi-task AE model.

A multi-task AE is developed and its network architecture is shown in Figure 5. The encoder takes *I*_*k*_ *(k*=*1, …, K)* as its input and outputs a one-dimensional vector *h* in a bottleneck. It consists of four down conv blocks and one linear block. Each down conv block consists of one 3 × 3 convolution by strides of two and one 3 × 3 convolution by strides of one, each followed by a batch normalization layer and a rectified linear unit (ReLu). Each linear block consists of a linear layer followed by a ReLu.

There are two decoders specified for two reconstruction tasks: 2D aperture map *A*_*k*_ and dose *D*_*k*_. For the first decoder, as shown in the upper side of Figure 5, the decoder takes the 1D vector *h* as input and reconstructs the 2D aperture map *A*_*k*_. It includes a linear block with the same structure as one of the encoders and four up-conv blocks. Each up-conv block consists of one up-convolution by strides of two and one convolution by strides of one, each followed by a batch normalization layer and ReLu. For the second decoder, as shown in the lower side of Figure 5, the decoder takes the 1D vector *h* as input and reconstructs the single dose *D*_*k*_. It contains three linear blocks with the same structure as the one in the encoder.

For reconstructing the aperture map in the first decoder, the binary cross-entropy loss *L*_*A*_ between the original apertures *A*_*k*_ *(k*=*1, …, K)* and the reconstructed apertures *A*_*k*_'* (k*=*1, …, K)* is minimized as defined below,


(3) $$\begin{array}{l} L_{A}\left(A,\;A^{\prime }\right)=\frac{1}{K\times M\times N}\overset{K}{\underset{k}{\sum }}\overset{M}{\underset{i}{\sum }}\overset{N}{\underset{j}{\sum }}[A_{kij}\log A_{kij}^{\prime }\\ \;+\left(1-A_{kij}\right)\log\left(1-A_{kij}^{\prime })\right], \end{array}$$

where *A*_*kij*_ and ${A^{\prime }}_{kij}$ are the pixels of the original *A*_*k*_ and $A_{k}^{\prime }$ at *k*-th control point, and *M* and *N* are the dimensions of the aperture map.

For reconstructing the dose in the second decoder, the mean-square error *L*_*D*_ is used to penalize the distance between the original dose *D* and the reconstructed dose *D*′ as defined below,


(4) $$\begin{array}{l} L_{D}\left(D,\;D^{\prime }\right)=\frac{1}{K}\overset{K}{\underset{k}{\sum }}{\left[D_{k}-D_{k}^{\prime }\right]}^{2}, \end{array}$$

where *D*_*k*_ and $D_{k}^{\prime }$ are the original dose and the reconstructed dose at the *k*-th control point. These two loss functions are weighted by parameter λ and form the overall loss function *L*_*R*_ of multi-task AE as defined below:


(5) $$\begin{array}{l} L_{R}=\lambda L_{A}\left(A,\;A^{\prime }\right)+L_{D}\left(D,\;D^{\prime }\right), \end{array}$$

where λ is the weighting factor. During the model learning, the reconstruction error *L*_*R*_ is minimized using the Adam optimizer with a learning rate of 1e-3.

### 2.3 Model learning

**Figure 6:**
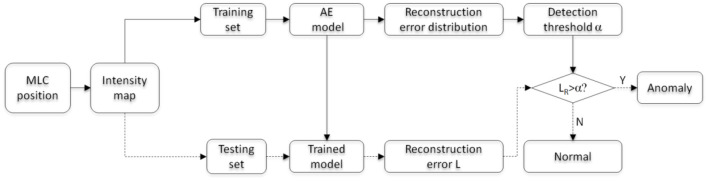
Flowchart of model learning and testing.

The learning and testing process of the multi-task AE model is illustrated in Figure 6. The workflow of model learning and model testing are labeled with solid and dashed lines. The model learning is first performed on the training set. Then, the trained model is validated on the testing set.

For evaluating the model performance, three distance metrics were employed to measure the difference between the reconstructed and original outputs. The first metric measures the distance between the original *I* and the reconstructed *I*′ as defined below:


(6) $$\begin{array}{l} ||I,\;I^{\prime }||=\frac{1}{K}\overset{K}{\underset{k=1}{\sum }}{\left[I_{k}-{I^{\prime }}_{k}\right]}^{2}, \end{array}$$

where *K* is the total number of CPs. In addition to *||I, I*′*||*, the other two metrics are the distance *||A, A*′*||*, which measures the binary cross-entropy between the original *A* and the reconstructed *A*′ as *L*_*A*_ defined in Equation 3, and the distance *||D, D*′*||*, which measures the distance between the original *D* and the reconstructed *D*′ as *L*_*D*_ defined in Equation 4.

The model is first learned using all feature maps of the training set. Then, the distribution of reconstruction error *L*_*R*_ for all training samples is obtained. To ensure all anomalous plans are detected and the least regular plans are falsely detected, the maximum value causing 0 false negative rate (FNR) is chosen as the detection threshold value α. For a testing sample, the reconstruction error *L*_*R*_ is obtained with the trained AE model and compared with the threshold α. If *L*_*R*_ is more than the threshold α, this plan is classified as an anomalous plan; otherwise, it is a regular plan.

In clinical scenarios, the learned model could be integrated into the oncology information system (OIS). For each plan review task, the feature vector of a VMAT plan will be extracted and fed to the model for a decision. If an anomaly is detected, the plan will be sent back to the planner for revision. After revision, it will be re-examined by the AI model until it passes the examination. Since a semi-automated anomaly detection module already exists in the current system, implementing this AI model would be feasible by following the footprint of the existing semi-automated module, with a similar function and interface.

### 2.4 Evaluation

All treatment plans in this study are VMAT plans. Each plan consists of two arcs, and each arc has 90 CPs (a total of 180 CPs for two arcs). These plans are executed on Synergy (Elekta Oncology Systems, Crawley, UK), equipped with 40 pairs of MLC leaves. The MLC leaves are installed on two banks, and the leaf width is 1.0 cm. The maximum gap width formed by these MLC leaves is 400 mm. A total of 677 VMAT plans for lung cancer patients treated in our institute between 2010 and 2020 years were used in this study. These plans are typical VMAT configurations designed with two full arcs and a beam energy of 6 MV. Among them, 652 plans are regular and 25 plans are anomalous. For model learning, 80% of the regular plans are assigned to the training set, while the remaining 20% of the regular plans, along with all anomalous plans are assigned to the testing set. Five-folder cross-validation is performed for model evaluation. The models' performance is mainly evaluated based on the area under the receiver operating characteristic curve (AUC). In addition, the other statistics such as false positive rate (FPR), accuracy, precision, and *F*1-score are also evaluated.

ROC shows the ability of the model to distinguish between anomalous and regular plans, and AUC is the area under the ROC. The classification model with a larger AUC means better anomaly detection capability. FPR [false positive/(false positive + true negative)] judges a case as abnormal when it is normal. The accuracy [(true positive + true negative)/(true positive + false positive + true negative + false negative)] and precision [true positive/(true positive + false positive)] of the model were calculated to evaluate the performance comprehensively. In addition, considering the highly unbalanced distribution of abnormal and normal classes in the dataset, the *F*1 score [2 ^*^ precision ^*^ recall/(precision + recall), where recall = true positive/(true positive + false negative)] of the model was also calculated. Since anomalous plans may cause irreversible damage to patients, all the metrics except AUC are evaluated while maintaining a false negative rate (FNR) to 0 (to ensure no anomalous plans are missed).

To evaluate the sensitivity of the distance metrics on the detection accuracy of anomaly, three types of distances are tested. They are the three distance metrics as defined in Equations 3, 4, 6. To compare the proposed model with the other existing AE models, three AE models, including Vanilla AE, Contractive AE, and Variational AE, are evaluated. Vanilla AE is a simple AE with a single encoder and decoder, and a mean-square error-based loss function is used to penalize the distance between the original and reconstructed inputs (Kingma and Welling, 2014). Contractive AE (CAE) is another kind of improved AE to learn robust features by introducing the Frobenius norm of the Jacobian matrix of the learned feature with respect to the original input (Michelucci, 2022). Rather than building an encoder that outputs a single value to describe each latent state attribute, Variational AE (VAE) provides a probabilistic manner to describe an observation in latent space (Aamir et al., 2021). To fairly evaluate the performance of all models, only *||I,I*′*||* is used as the distance metric.

## 3 Results

### 3.1 Reconstruction error

The anomaly detection accuracy of the model with respect to the three distance metrics is shown in Table 2. The multi-task AE with the metric ||*I,I*′|| achieved the best performance with an AUC value of 0.964. While maintaining 0 FNR, the accuracy and precision of the model with the metric ||*I,I*′|| are 0.821 and 0.471, respectively. The accuracy and precision are 0.314 and 0.185 for metric the ||*A,A*′||, while they are 0.365 and 0.197 for metric the ||*D,D*′||. The detection performance of multi-task AE with metric the ||*I,I*′|| is the best among the three forms of distances.

**Table 2:** Performance of the multi-task AE model with respect to the three distance metrics.

**Distances**	**AUC**	**Accuracy**	**Precision**	**FPR**	***F*1 score**
||*I,I′*||	0.964	0.821	0.471	0.206	0.632
||*A,A′*||	0.819	0.314	0.185	0.809	0.310
||*D,D′*||	0.855	0.365	0.197	0.748	0.327

**Figure 7:**
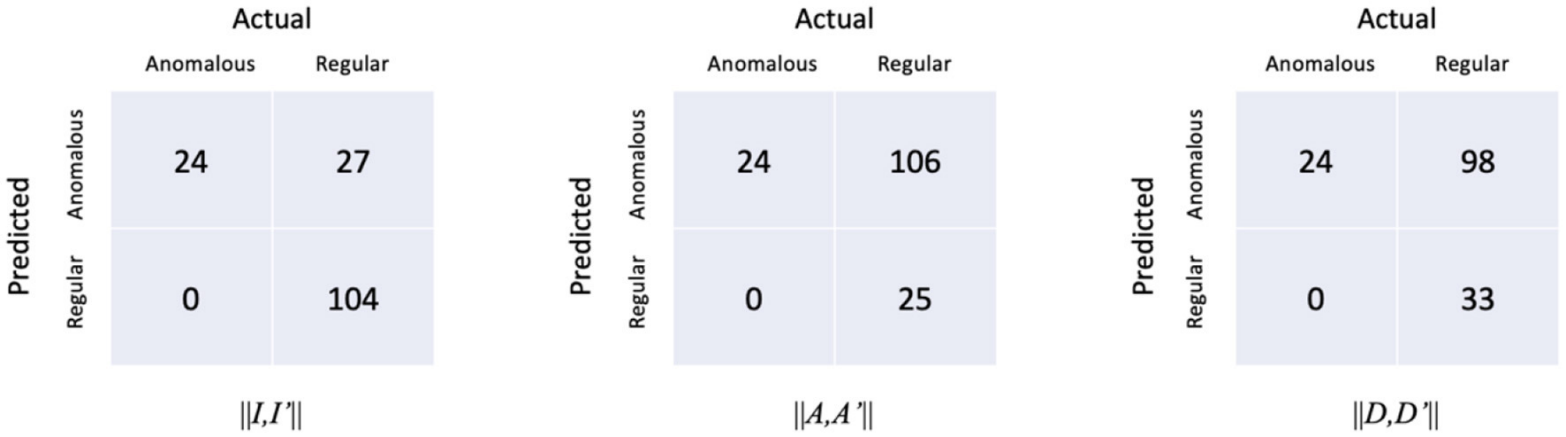
Confusion matrices of the multi-task AE model with respect to the three distance metrics.

The confusion matrices of the proposed model with respect to the three distances are shown in Figure 7. It is worth noting that all the anomalous plans were correctly detected since the confusion matrix is achieved with 0 FNR. The model with the distance metrics *||I,I*′*||* misclassified 27 regular plans as anomalous plans. While the model with the distance metrics *||A,A*′*||* and *||D,D*′*||* misclassified 106 and 98 regular plans as anomalous plans. The multi-task AE with the distance metric *||I,I*′*||* had the best detection accuracy.

**Figure 8:**
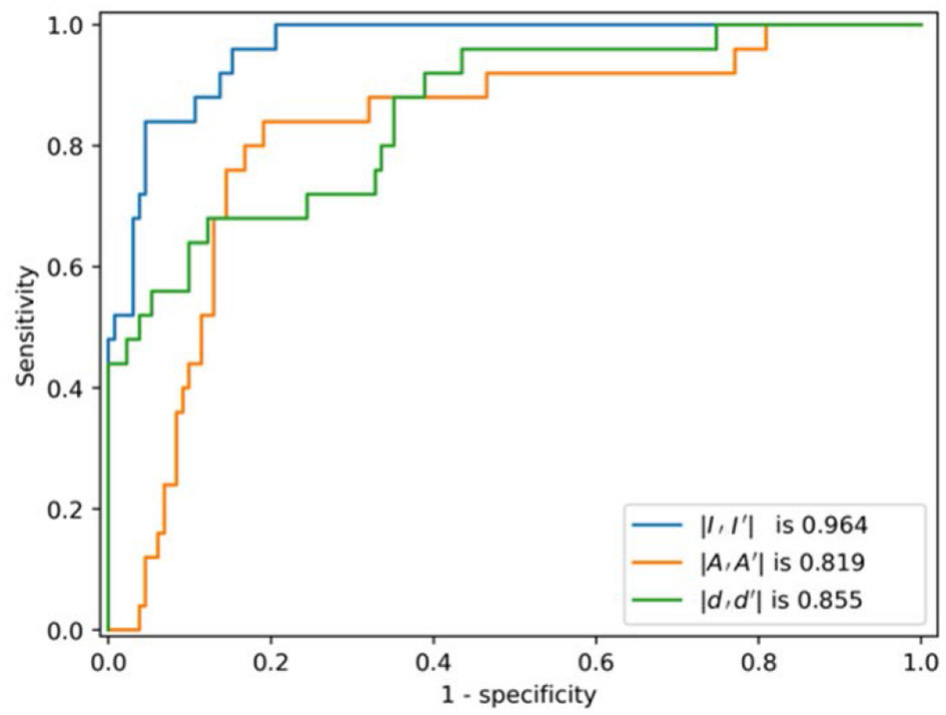
ROCs of the model with respect to the three distance metrics.

The ROC curves of the multi-task AE model with respect to the three distance metrics are compared in Figure 8. The AUC of the multi-task AE with the metric ||*I,I*′|| is the highest. The AUC of the multi-task AE with the metric || *A,A*′|| is lower than that with metric ||*I,I*′|| but higher than that with the metric ||*D,D*′||. The multi-task AE models, with three types of distances, all achieve higher AUC values (more than 0.8), which indicates it is advantageous to apply 2D intensity maps for anomaly detection in VMAT plan review.

**Figure 9:**
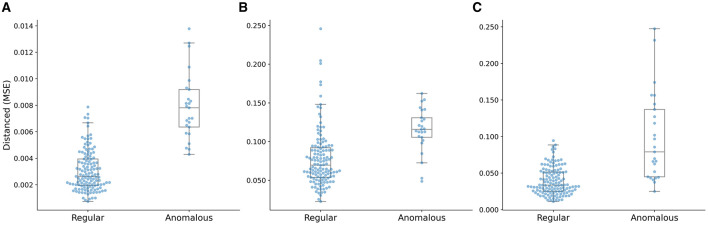
Distance distributions of the multi-task AE model with three distance metrics. **(A)** ||*I,I*′||, **(B)** ||*A,A*′||, and **(C)** ||*D,D*′||.

The box plot of the distance distributions of the multi-task AE model with respect to the three distance metrics is shown in Figure 9. The top and bottom horizontal lines indicate the maximum and minimum values of the distribution, respectively. The points beyond these two lines are outliers. The top and bottom edges of the box denote the 75th and 25th percentiles of the distribution, respectively. The middle line in the box represents the median value of the distribution.

The median value of the distribution of the anomalous plans is higher than the maximum value of the distribution of the regular plans, and the minimum value of the distribution of the anomalous plans is close to the 75th percentile of the distribution of the regular plans, as shown in Figure 9A. The median value of the distribution of the anomalous plans is lower than the maximum value of the distribution of the regular plans, and the minimum value of the distribution of the anomalous plans is lower than the median of the distribution of the regular plans as shown in Figure 9B. The median value of the distribution of the anomalous plans is lower than the maximum value of the distribution of the regular plans, and the minimum value of the distribution of the anomalous plans is lower than the 25th percentile of the distribution of the regular plans as shown in Figure 9C. The gap between the clusters of the anomalous and regular plans on the distance distribution of the multi-task AE model with ||*I-I*′|| is the largest.

### 3.2 Model comparison

**Figure 10:**
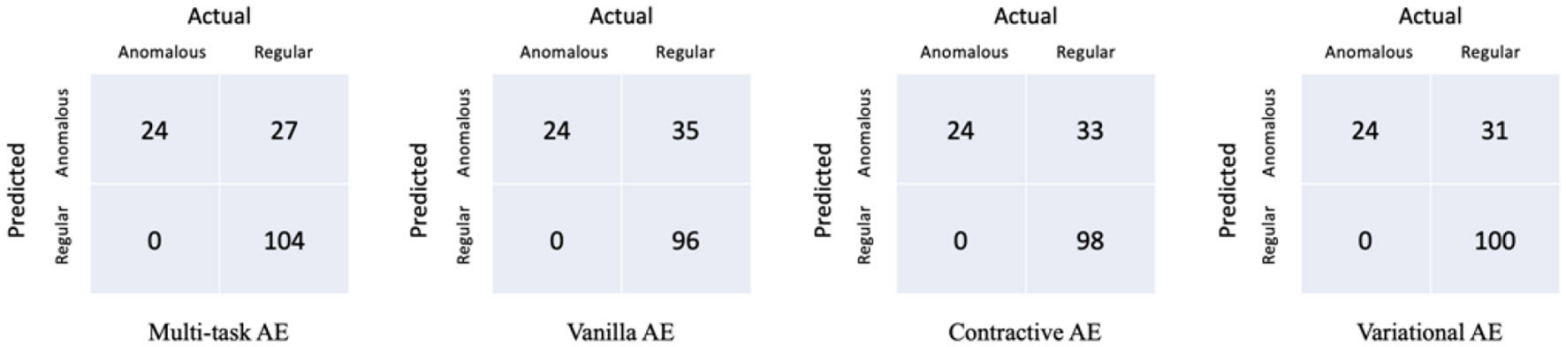
Confusion matrices of the four models with the distance metric *||I,I*′*||*.

The performances of the multi-task AE and three existing AE models are compared in Table 3. The confusion matrices of the four AE models with the three distance metrics ||*I,I*′|| are shown in Figure 10. The multi-task AE achieved the best performance, while Variational AE and Contractive AE are comparable and slightly lower. Vanilla AE has the lowest AUC score but is higher than 0.93. While maintaining 0 FNR, the accuracy and precision of multi-task AE are both the highest among the four models. The accuracy and precision of Variational AE and Contractive AE are comparable and slightly lower. The accuracy and precision of Vanilla AE are the lowest among the four AE models.

**Table 3:** Performance of four AE models with the distance metric ||*I,I*′||.

**AE models**	**AUC**	**Accuracy**	**Precision**	**FPR**	***F*1 score**
Multi-task AE	0.964	0.821	0.471	0.206	0.632
Vanilla AE	0.943	0.769	0.407	0.267	0.571
Contractive AE	0.936	0.782	0.421	0.252	0.585
Variational AE	0.948	0.795	0.436	0.237	0.600

**Figure 11:**
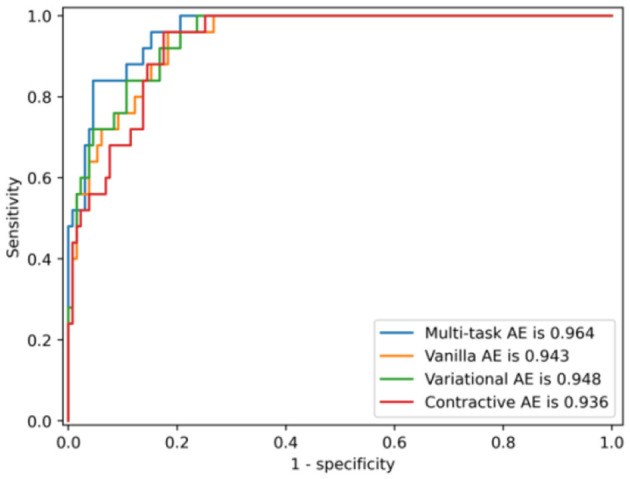
ROCs of four AE models with the distance metric ||*I,I*′||.

The ROC curves of the four AE models with the distance metric ||*I,I*′|| are compared in Figure 11. The AUC of the multi-task AE is the highest. The AUC of Variational AE and Contractive AE is comparable and lower than that of multi-task AE. The AUC of Vanilla AE is the lowest. All AE models have AUC scores of more than 0.94, which indicates it is advantageous to apply 2D intensity maps for anomaly detection in VMAT plan review.

**Figure 12:**
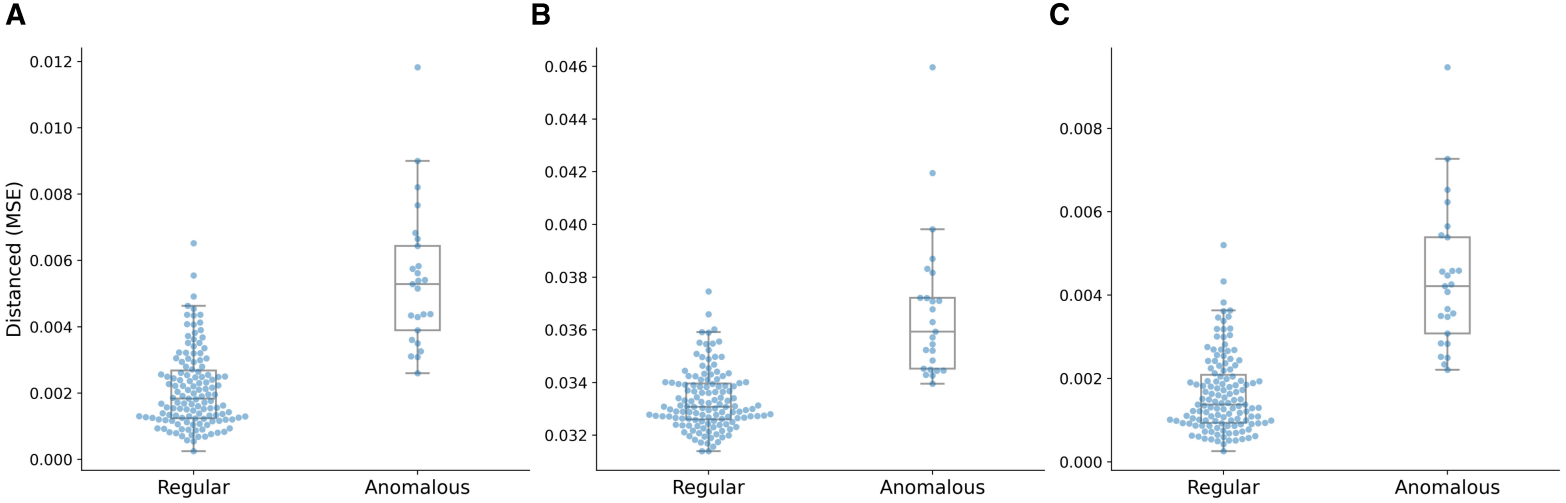
Distance distributions of three AE models with the distance metric ||*I,I*′||. **(A)** Vanilla AE, **(B)** Contractive AE, and **(C)** Variational AE.

The box plot of distance distributions of three AE models with the distance metric ||*I,I*′|| is shown in Figure 12. The median value of the distribution of the anomalous plans is close to the maximum value of the distribution of the regular plans, and the minimum value of the distribution of the anomalous plans is lower than the 75th percentile of the distribution of the regular plans, as shown in Figure 12A. The median value of the distribution of the anomalous plans is lower than the maximum value of the distribution of the regular plans, and the minimum value of the distribution of the anomalous plans is close to the median of the distribution of the regular plans, as shown in Figure 12B. The median value of the distribution of the anomalous plans is higher than the maximum value of the distribution of the regular plans, and the minimum value of the distribution of the anomalous plans is close to the 75th percentile of the distribution of the regular plans, as shown in Figure 12C. The distance distribution of Variation AE is closer to that of the multi-task AE model, as shown in Figure 9A. This result is also consistent with those shown in Table 3 and Figure 11.

## 4 Discussion

This study evaluates the performance of multi-task AE and the other three classic AE models in detecting anomalies from routine radiotherapy plans using the MLC aperture-based feature map. In contrast to the discrete geometry features such as distance and angle, the 2D aperture-based feature map provides not only the beam shape but also the dose information. In addition, as a set of feature maps is created for all CPs and subsequently used for model learning, the leaf positions at adjacent CPs can be checked to ensure their consistency for plan delivery. Our study is different from the heatmap proposed by Kump et al. (2022), in which the 2D intensity map is generated based on the summation of a set of intensity maps for all CPs in an IMRT plan. The heatmap may be sensitive in distinguishing IMRT plans with different treatment sites but may be insufficient in characterizing the leaf position relationship at the adjacent CPs in the VMAT plan. As the VMAT plan consists of hundreds of CPs, which is more complex than the IMRT plan, a set of 2D feature maps is more useful to characterize the leaf movement than the heatmap.

Compared to the classic machine learning method such as PCA, which represents high-dimensional plan features with low-dimensional linear features, the proposed AE model is more effective. The AE model uses a non-linear activation function in the encoder/decoder, allowing the neural network to arbitrarily approximate any non-linear function. This allows the network to learn more complex mapping relationships between high-dimensional space and low-dimensional space to better fit the distribution of normal data and thus to find anomalous data with a very small percentage through the network. In addition, the proposed model uses two outputs to represent two key features of a beam, which makes the model more sensitive to the anomalous plan. The AUC scores also show that the proposed AE model outperforms the other existing AE models in addition to having the highest accuracy and precision.

The advantage of the multi-task AE model is its dual outputs with regard to two critical parameters of a VMAT plan: aperture and dose. We can easily calculate the distance between the original and the reconstructed values for aperture and dose, respectively. This is convenient as we can detect anomalies by focusing on the specific features: leaf position or dose. For achieving this goal, there are certain tradeoffs in the model learning and detection processes. First, the loss function used in model learning is Equation 5 instead of Equation 6, which takes more time to compute two loss functions (*L*_*A*_ and *L*_*D*_). If the reconstructed intensity map is needed, its value has to be calculated from the reconstructed aperture and reconstructed dose as defined in Equation 6. Since three distance metrics can be calculated from the output of the multi-task AE model, it is more flexible than the other AE models with a single output.

Applying an intensity map with a multi-task AE model in a radiotherapy plan review is promising, but there are several aspects to be improved. First, only VMAT plans for lung cancer patients are used in this study. More treatment sites will be included and tested using current models. In addition, due to the limitations of the paper, the effectiveness of this model on the other treatment modalities is not validated and should be performed in the future. Second, the components of the multi-task AE model used in this study are relatively simple. This model can be improved by introducing attention and adversarial mechanisms. In addition, parameter tuning is also another challenge for the deep-learning model. Third, the numbers of the regular and anomalous plans are severely imbalanced. This could result in models that have poor predictive performance, specifically for the minority class (anomalous plan in this study). To alleviate this issue, synthetic data generated using GAN-based techniques should be used to compensate the minority class in future studies.

The proposed multi-task AE model utilizes the reconstruction error to classify a plan as anomalous or regular and exhibits excellent performance. However, the model is not able to provide additional information on how to improve or modify the plan in response to the anomalous event. In a clinical setting, it is critical to understand the rationale behind decisions, and therefore an explainable AI model is needed (Caroprese et al., 2022). In this study, it is also helpful to determine the cause of anomalies with a transparent AI model. To address this issue, the plan representations in latent space should be partitioned meaningfully into several semantic regions, allowing for the identification and correlation of the underlying causes of any anomalies. It would be possible to implement this idea using an adversarial autoencoder (Schreyer et al., 2019), which provides a holistic and semantic view of plan representations in latent space. Combining our model with AAE would be promising for future research.

## 5 Conclusion

The aperture-based intensity map provides a simple way to characterize the shapes in the VMAT plan. The proposed AE model is more accurate in detecting anomalies from routine radiotherapy plans compared to the existing deep-learning models. The combination of feature maps and the multi-task AE model provides an effective way to perform automated plan reviews for VMAT plans. The multi-task AE model could also be used in a plan review of the other types of plans with different treatment sites and modalities. It is also promising to combine the explainable AI with the current model for a more clinically interpretable anomaly detection model for current VMAT plan reviews in radiotherapy.

## Data Availability

The original contributions presented in the study are included in the article/supplementary material, further inquiries can be directed to the corresponding authors.
